# Supplementary material for: Patterns of Intron Gain and Loss in Fungi
Source: PLoS Biol. 2004 Nov 30;2(12):e422. doi: 10.1371/journal.pbio.0020422 (PMC532390; doi:10.1371/journal.pbio.0020422)
Supplement: Table S1 — Also available at http://genes.mit.edu/NielsenEtAl/. (4.3 MB ZIP). [file pbio.0020422.st001.zip › NielsenEtAl/html/645.html]

AN0770.1.NCU01928.1.MG02940.1.FG08927.1


```
 CLUSTAL W (1.82) Multiple Sequence Alignments - Introns Inserted


Sequence 1: NCU01928.1	514 aa
Sequence 2: FG08927.1	525 aa
Sequence 3: MG02940.1	512 aa
Sequence 4: AN0770.1	512 aa
Alignment Length: 545 aa
Number Identitical Residues: 174 aa
Alignment Score (without introns) 9848


MG02940.1 	-MADGEPDISDLSPEQQEALQQYTMITDQSVQDAIPLLQRSQWNAQ0IAIAKFFDGEQPD
NCU01928.1	MSGDNAVDIGQLSPEQQQALQQYTDVTGQEITDAIPFLERSQWNVQ0IAIAKFFDGEGPD
FG08927.1 	-MDESEVDLGQLSSTQQEALNQYTQVTNQEINEAIPLLRRSEWNVQ0IAIAKFFDGETAD
AN0770.1  	-MSSSGPDISELSDSQRSALETYTTVTGQEPVEAIALLSRSQWNVQ0IAIAKFFDGEGPD
          	   ..  *:.:**  *:.**: ** :*.*.  :**.:* **:**.* ********** .*

MG02940.1 	PVAEALAAQEQNQQAGGPGLGGTARFENLQDSLDG-IEMDIVRSRRHRTEAAPRVVPQP-
NCU01928.1	LVAEAQAAQNQVPRVAG-------RHETLHETVWSDIAHQHGLHRANRTPPAPRVVPPR-
FG08927.1 	PIA---AAQQEIPRATA-------RHENLQESLFHEAARPSRASPRQRTDLAPRIVPQN-
AN0770.1  	PVEEARASLESASPARP---------NRQTQNLLHEDLTARFSSASAAADPAPRIATQPG
          	 : .: *: :.   .           :   :.:  .            :  ***:..  .

MG02940.1 	-ARIYRVPFLWSIITLPFSLTWRIATTLFRPIVYLLGLLPCNMRPRAIG---AAGYG---
NCU01928.1	-PVTYQAPSLISFLLSPFRMVLRVFASLFRPILYILSFIPQSLRPHALT----ASFR--K
FG08927.1 	-SVTNRAPWLLGLLLTPFSLGWRAASTLFRTLLYALSFLPASIRPRAVTSRISTGFRGTN
AN0770.1  	DQPVYRPPFLLALVFAPFNLLYRLFLGSFRLFGSLFPFLPRLLNTTASPALQGVGRN-TN
          	.    : * * .::  ** :  *     ** :   : ::*  :.. *  :  ...   :.

MG02940.1 	-RRQLMPRDAAARFKREFEENYGSGELPWYDGGSAQAYDAAKRELKFLLIVLLSPEHDDT
NCU01928.1	SRRSLLPKETAGRFKREFEEYYGTHDLVFFDGGHAQALDTAKKDLKFLLTILISPEHDDT
FG08927.1 	GRRPLMPRDTASRFKREFEEEYGENDLPFFEGGVAQAHDQAKKDLKFMLVVILSPEHDDT
AN0770.1  	GRRPLAPKDTAARFIREFEEEYGAHSLPFLENGYNMALEKAHRDLKFLLVVLCAPEHDHT
          	.** * *:::*.** ***** **  .* : :.*   * : *:::***:* :: :****.*

MG02940.1 	DSFVRDVLLSPEVVSFIKDEKNNIILWGGNIVDSEAYQVSFEYRCTRFPFACLVCLTPKE
NCU01928.1	DSFIKDTLLDPEVVAFINDPANNIIIWGGNVLDSEAYQVSMEYTCTKFPFSCLVCLTPKE
FG08927.1 	ESFVKETLLSPEVVAFIKDPSNNILLWGGNVLDSEAYQVAQEYICTKFPFSALVCLTPKE
AN0770.1  	DAWVRETLLSKEVTDFINDSQNNIIVWGGNVQDAEAYQVANSIRCTKFPFAAAIAHTPSV
          	::::::.**. **. **:*  ***::****: *:*****: .  **:***:. :. **. 

MG02940.1 	GSTRMGTIKRIQGAVTPERFLGDIRSAIHKHNPDLDGVRAERVAQEAARNLRADQDSAYE
NCU01928.1	GSTRMGIVKRIAGPVSPSVFIAGIRGAIEKYAPDLDSVRAERAAQDMARNLRSEQDSAYE
FG08927.1 	GSTRMGIVKRLVGPMPSATYLSEIRAAIEKYGADLDGVRAERTAQEVTRNLRTEQDSAYE
AN0770.1  	SSTAMSVIARISGVTSPAEFVEKLRTAIAQNKEPLERIRVTRAEQQASRSLREQQDSAYE
          	.** *. : *: *  ..  ::  :* ** :    *: :*. *. *: :*.** :******

MG02940.1 	RSLAKDREKARLRREAEAAAAAAEKRALEEAEAAARLEEKRRQWKAWRATTLAEEPPAAE
NCU01928.1	RSLAIDRERARQRREAAAAAAEAERRAREEAEAAERKEKLRQQWRRWRATTIAAEPDVSV
FG08927.1 	RSLAIDRERARQKKEAAAAAAAAEKRALEEANAAAILEEKRWKWKAWRAATIVAEPPTSE
AN0770.1  	RSLAIDRERARQRREAEAARQREEQLAAERQAAEEKRQRDLEQWKRWRAQSIPEEPDMGV
          	**** ***:** ::** **    *: * *.  *    :.   :*: *** ::  **  . 

MG02940.1 	KDVVRLALMMPEYTGAGRIVRRFARETTMEELYAFVECYDATRGDA-------EKPPLQP
NCU01928.1	KDAVRLALNMSQSSGRGRVTRKFAPDASLEDVYAFVECYDLLYPEDEEEKGDVEESTDKP
FG08927.1 	KNVVRVALKMPEESGIGRIVRRFPQDASLEDMYAFVECYSVLRQDD-------AAVDDRP
AN0770.1  	KDAVRISIRLP--SG-DRVIRRFAPDAVMEELYAFVECYDITKEAEAAT----PTPVEKP
          	*:.**::: :.  :* .*: *:*. :: :*::*******.                  :*

MG02940.1 	DDYEHEYKFRIVSPMPREVYEPSTAATVGDKIGRSGNLIVETIVS----------DSDDE
NCU01928.1	ENYEHKYAFRIASVMPREVFEPTASVTIAQKMGRGGNLIVEDL-----------VDEEEE
FG08927.1 	EDYEHEYKFRIASILPREVYEPSTTVTMGEKIGRSGNLIVEDVSLDSEDESDDDVDDEDE
AN0770.1  	GGYDHTYGFRLVSPMPRVVYAVDAG-SIRDNVGRGGSLLVEPID-----------DESDE
          	 .*:* * **:.* :** *:   :  :: :::**.*.*:** :            *..:*

MG02940.1 	E-----
NCU01928.1	E-----
FG08927.1 	ETEAVQ
AN0770.1  	EDS---
          	* .
```
